# Structured assessment of brain MRI in Covid-19–related neurological disease: an international multicentre study

**DOI:** 10.1007/s00234-025-03787-8

**Published:** 2025-09-26

**Authors:** Stéphane Kremer, Tarek A. Yousry, Rafael Rehwald, François Lersy, Nicolas Meyer, Simonetta Gerevini, Angela Napolitano, Àlex Rovira, Ng Karelys, Leandro Tavares Lucato, Maria da Graça Morais Martin, Ana Lícia da Rocha Alves Pinto, Luke Dixon, Brynmor Jones, Ana Ramos, Elena Salvador, Núria Bargalló, Laura Oleaga, Nicoletta Anzalone, Andrea Falini, Francesco Carletti, Chandrasekhar Hoskote, Agathe Chammas, Benoit Lhermitte, Béatrice Lannes, Thibaut de Misouard, François Cotton, Hans Rolf Jäger

**Affiliations:** 1 Hôpitaux Universitaires de Strasbourg, Service d’imagerie 2, Hôpital de Hautepierre, Strasbourg, France; 2 Engineering Science, Computer Science and Imaging Laboratory (ICube), Integrative Multimodal Imaging in Healthcare, UMR 7357, University of Strasbourg-CNRS,, Strasbourg, France; 3 Neuroradiological Academic Unit, UCL Queen Square Institute of Neurology, London, United Kingdom; 4 Lysholm Department of Neuroradiology, The National Hospital for Neurology and Neurosurgery, London, United Kingdom; 5 CHU de Strasbourg, Service de Santé Publique, GMRC, Strasbourg, France; 6 Neuroradiology Unit, ASST Papa Giovanni XXIII, Bergamo, Italy; 7 Neuroradiology Section, Hospital Universitari Vall d’Hebron, Barcelona, Spain; 8 Neuroradiology Section, Hospital das Clínicas, Faculdade de Medicina, Universidade de São Paulo (HCFMUSP), São Paulo, Brazil; 9 Department of Neuroradiology, Imperial College Healthcare, NHS Trust, London, United Kingdom; 10 Sección de Neuroradiología, Hospital Universitario 12 de Octubre, Madrid, Spain; 11 Hospital Clínic de Barcelona, Barcelona, Spain; 12 Department of Neuroradiology, IRCCS San Raffaele, Scientific Institute and Vita-Salute San Raffaele University, Milan, Italy; 13 Lysholm Department of Neuroradiology, The National Hospital for Neurology and Neurosurgery, London, United Kingdom; 14 Hôpitaux Universitaires de Strasbourg, Service d’Anatomopathologie, Hôpital de Hautepierre, Strasbourg, France; 15 MRI center, Centre Hospitalier Lyon Sud, Hospices Civils de Lyon, Lyon, France; 16 Observatoire Français de la Sclérose en Plaques, Bron, France

**Keywords:** Covid-19, MRI, Brain, structured reporting, Classification

## Abstract

**Purpose:**

Neuroradiological findings associated with neurological presentations in acute SARS-CoV-2 infection are very heterogeneous. We aimed to develop a standardized framework for describing MR neuroimaging patterns in Covid-19, to test this in an international multicentre study and to determine the prevalence of observed MRI patterns and their association with clinical presentation and outcome.

**Methods:**

An international expert consortium developed a framework for assessment of brain MRI patterns in Covid-19 based on published literature and professional experience. We performed a retrospective analysis of the proposed framework, involving brain MRI scans from 458 Covid-19 patients with neurological symptoms, including data from 1 February to 31 May 2020. Two readers at 25 centres across five countries assessed the local MRI studies regarding the presence of one or more predefined MRI patterns. Imaging and clinical data were analysed using Bayesian statistics.

**Results:**

Of 458 patients, 58.5% had an abnormal MRI. Overall, 94% of all imaging pathologies seen were captured by our proposed classification. Ischemic strokes were the most frequent pattern overall (25.6%), followed by microhaemorrhages (15.9%). Ischemic infarct patterns were more frequent in non-ICU patients, while the haemorrhagic patterns were more frequent in ICU patients. White matter lesions (10.9%) were more frequent than grey matter lesions (8.1%), and leptomeningeal contrast enhancement was present in 8.3% of patients. Patient outcome was not associated with any MRI patterns.

**Conclusion:**

Our proposed classification of specific MRI patterns in Covid-19, covered 94% of observed abnormalities, while patient outcome, death or home discharge, was not associated with any MRI patterns.

**Supplementary Information:**

The online version contains supplementary material available at 10.1007/s00234-025-03787-8.

## Introduction

Following the initial report of neurological manifestations in patients with Covid-19 [1], their broad clinical spectrum became rapidly clear [2, 3], ranging from mild symptoms, such as headache or anosmia, to severe conditions, including encephalopathy and impaired consciousness [2, 4]. The precise incidence and prevalence of neurological involvement in Covid-19 during the first wave of the pandemic are however difficult to assess [5]. Neurological manifestations appear more frequently in patients with severe Covid-19 infection [2] and Covid-19–associated encephalopathy seems to carry a poorer prognosis and a higher mortality [6].

Different hypotheses have been suggested regarding the underlying pathophysiological mechanisms [2, 7–9], including direct neurotropic infection [10] via different pathways [11] or indirect damage due to exaggerated activation of the immune system, also known as “cytokine storm”, coagulopathies, systemic hypotension, hypoxemia [12, 13], electrolyte imbalance, as well as post-infectious immune disorders [14, 15].

Direct neurotropic infection is probably the least common mechanism of neurological involvement in Covid-19, as the SARS-CoV-2 virus is rarely detected in cerebrospinal fluid and has been documented in only a limited number of studies in brain tissue [14, 16].

Neuropathological studies have demonstrated a considerable heterogeneity of cerebral lesions, (eTable 1 in the electronic supplementary material) [17] with three primary mechanisms of damage identified: hypoxic changes, thrombotic complications, and acute disseminated encephalomyelitis (ADEM)-like immune responses.

Similar to the clinical manifestations and neuropathological findings in Covid-19, the neuroradiological patterns described in the literature are also heterogenous [4, 18–21] (eTable 2), ranging from acute ischemic strokes and intraparenchymal haemorrhages, variable patterns of intraparenchymal lesions affecting the white matter, grey matter or both, to leptomeningeal enhancement (eTable 2).

The prevalence of the different neuroimaging patterns and their association with neurological symptoms and outcome or severity of Covid-19 infection remains largely unknown, considering the heterogeneity of available data and the limited number of patients included in individual studies.

This work aims to develop and propose a reproducible, standardized framework to describe different neuroimaging patterns associated with Covid-19 based on an extensive review of the literature and expert consensus. We aim to retrospectively test and implement the proposed framework in a large-scale, international multicentre study in MRI scans acquired during the first wave of Covid-19, examining the prevalence of Covid-19–associated MRI patterns, their associated clinical presentations, and patient outcomes.

## Materials and methods

This study is an international multicentre, retrospective, observational neuroradiological study among patients with confirmed Covid-19 presenting with neurological symptoms who underwent clinical brain MRI.

### Patient cohort

The study cohort comprised 458 patients with Covid-19 in 25 centres from five different countries: Brazil, France, Italy, Spain, and the United Kingdom (eTable 3). The mean age was 62.1 ± 14.7 years, 309 were men, and 149 women. Data were collected from 1 February 2020 to 31 May 2020.

The inclusion criteria were a case of Covid-19, defined as evidence of SARS-CoV-2 infection confirmed by reverse transcriptase-polymerase chain reaction (RT-PCR) assays on nasopharyngeal throat or lower respiratory tract swabs, or positive SARS-CoV-2 antibody serology; presenting with neurologic symptoms; and a diagnostic brain MRI performed for clinical reasons. The key exclusion criterion was missing or non-diagnostic MRI data (lack of basic sequences, degradation by artifacts).

### Brain MR imaging acquisition

Brain MR imaging was performed on 1.5 T or 3.0 T MRI systems. MRI scans were performed with a mean delay of 22.8 ± 18.9 days after, mainly respiratory, symptom onset (range [0; 120] days).

All MRI protocols included at least T2-weighted, FLAIR, DWI, and haemorrhage-sensitive sequences (99 T2*-weighted GRE and 342 SWI sequences). In 308 cases, an additional contrast-enhanced T1-weighted sequences were acquired, of which in 150 cases this were contrast-enhanced FLAIR sequences. Due to the retrospective multicentre design of the study, the MR imaging protocols employed at the participating institutions were not standardized.

### Brain MRI pattern categorization and image analysis

**Fig. 1:**
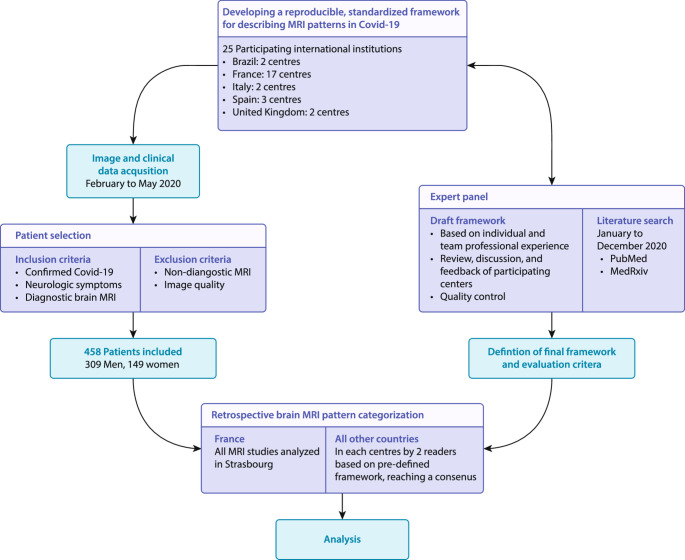
Study flowchart

An expert panel (SK, TAY, FC, HRJ) predefined a list of specific MRI patterns based on their professional experience and published literature on brain MRI findings in Covid-19 patients. The primary aim of our classification framework was to provide a structured system for categorizing brain MRI changes in COVID-19–related neurological disease. By standardizing terminology and lesion definitions, it enables accurate estimation of the prevalence of each pattern and its association with clinical presentation and patient outcomes. Additionally, by mapping the full spectrum of radiological findings (Fig. 1), it heightens clinician and radiologist awareness of both typical and atypical imaging patterns. To further define the MR imaging patterns, a literature search was conducted from 1 January 2020 to 31 December 2020 using the following keywords: “Covid-19 OR SARS-CoV-2” AND “brain OR encephalitis OR encephalopathy OR MRI OR neurology OR autopsy” in PubMed and medRxiv. The study selection and the data extraction are described in the supplemental materials (eMethods).

**Fig. 2:**
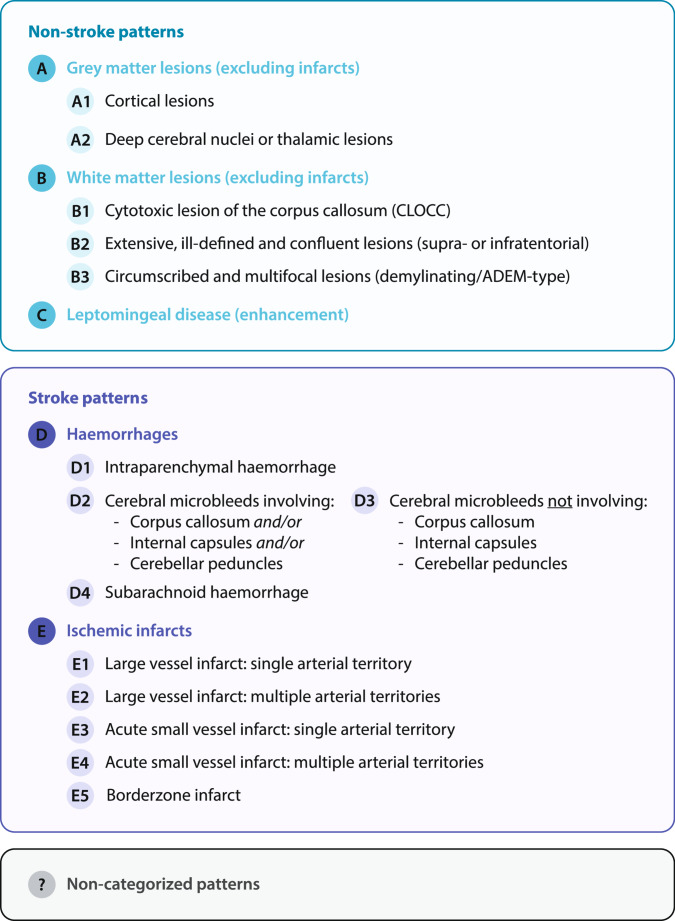
Proposed rating framework

The draft framework was designed to cover stroke and non-stroke patterns, including subgroups of lesions affecting the grey matter and white matter, leptomeninges, as well as haemorrhagic lesions and ischemic strokes. The initial classification was shared with all participating centres for review and discussion, integrating their feedback into the final version of the framework (Fig. 2). Except for the participating centres in France, where all examinations were centralized and analysed in Strasbourg, two readers at each centre (with an overall average of 19.3 ± 10.0 years of experience) independently assessed the local MRI studies. They reviewed all available sequences and images for each patient and reached a consensus on classifying the observed findings into one or more of the predefined categories. To account for imaging findings not covered by the predefined list, a category for “non-categorized patterns” was added.

### Clinical data

The following clinical data were extracted from the patients’ electronic health records: age, sex, SARS-CoV-2 RT-PCR test result, SARS-CoV-2 antibody test result, admission to the intensive care unit (ICU), oxygen supplementation, acute respiratory distress syndrome (ARDS), neurologic symptoms (headaches, seizures, anosmia, acute onset weakness, consciousness disorders, confusion, agitation), difficulties weaning from sedation, and patient outcomes. The diagnosis of ARDS was based on the Berlin criteria [22]. Patient outcomes were categorized as death or home discharge.

### Statistical analysis

After local analysis in each centre, the data were centralized at Strasbourg University Hospital and QS Institute of Neurology, London. The data were analysed using Bayesian statistics, allowing for a flexible and informative approach in the design, analysis, and interpretation of the data collected [23].

Continuous variables are reported as means and standard deviations, categorical variables are expressed as numbers and percentages. Results are given with their 95% posterior credible interval.

From a probabilistic point of view, the study aimed to calculate the probability that a proportion or mean difference was larger in either the ICU or non-ICU group. All priors were defined before the study.

The different categorical outcomes were modelled using logistic regression, which were then used to estimate proportions in each group. For each outcome, we calculated the odds ratio (not shown) and the proportion difference with the probability that the difference was larger than 10% in absolute value. This interval was considered to express a clinically relevant between-group difference. The log odds ratio was given a N(mean = 0, SD = 1.53) prior, such that the odds ratio lies a priori within the [0.05; 20] range with a probability of 0.95. A sensitivity analysis using a beta distribution of proportions yielded similar results for all categorical outcomes (not shown).

Age differences were estimated using normal distribution N(µ_G_, SD = 10), where µ_G_ was set to the observed mean age in each group G. The inverse of the variances was given weakly informative prior Gamma Ga(0.01, 0.01).

Missing data and death, hospitalization, rehabilitation, or home discharge were not imputed, and analyses for this outcome were performed on the non-missing dataset.

The posterior distribution characteristics were estimated using Markov chain Monte Carlo (MCMC) sampling, with a burn-in of 5,000 iterations, followed by 100,000 iterations for each of the two chains, and a thinning of 4, yielding a final 50,000-iterations sample. The convergence of the MCMC sample chains was evaluated graphically and with the Brooks–Gelman–Rubin test. Convergence was observed in each case. The absence of autocorrelation was also checked and was considered negligible in each case. All computations were performed with R versions 3.4.1 to 4.4.2 (https://www.r-project.org) and JAGS (https://mcmc-jags.sourceforge.io) with all required additional packages.

## Results

### Patient characteristics and outcomes

Patients were predominantly male (67.5%), hospitalized (96%) and mainly admitted to the ICU (57.4%), requiring oxygen supplementation (76%), and with ARDS (57.4%) (Table 1).

**Table 1:** Clinical characteristics and patient outcomes

	All patients (*N* = 458; 100%)	No ICU admission (*N* = 195; 42,6%)	ICU admission (*N* = 263; 57,4%)	Proportion difference with 95% Bayesian credible interval	Probability that the difference is > 10%
Sex, male	309 (67.5%)	116 (59.5%)	193 (73.4%)	13.6 [5.0; 22.2]	79.8%
Age (mean ± SD; range)	62.1 ± 14.7 [8–95]	66.5 ± 5 [10–95]	58.8 ± 13.5 [8–86]	−7.6 [−10.3; −4.9]	4%
Inpatients (at the time of MRI)	439 (96%)	177 (91%)	262 (99.6%)	7.9 [4.3; 12.1]	14.4%
Respiratory status					
*Oxygen supplementation*	348 (76%)	88 (45.1%)	260 (99%)	52.1 [45.0; 59.2]	100%
*Orotracheal intubation*	217 (47.4%)	8 (4%)	209 (79.5%)	73.7 [67.8; 79.2]	100%
*ECMO*	11 (2.4%)	0	11 (4%)	3.3 [1.2; 5.9]	0%
*ARDS*	263 (57.4%)	29 (14.9%)	234 (89%)	73.1 [66.5; 79]	100%
Neurological symptoms					
*Confusion*	183 (40%)	72 (36.9%)	111 (42.2%)	5.2 [−3.9; 14.1]	14.7%
*Consciousness disorders*	164 (35.8%)	37 (19%)	127 (48.3%)	28.8 [20.6; 36.9]	100%
*Acute onset weakness or aphasia*	126 (27.5%)	63 (32.3%)	63 (24%)	−8.2 [−16.5; 0]	33.6%
*Agitation*	92 (20%)	27 (13.8%)	65 (24.7%)	10.7 [3.6; 17.7]	58.1%
*Difficult weaning from sedation*	88 (19.2%)	4 (2%)	84 (31.9%)	28.8 [22.9; 34.8]	100%
*Headaches*	64 (14%)	36 (18.5%)	28 (10.6%)	−7.6 [−14.2; −1.3]	23.5%
*Seizures*	52 (25.3%)	22 (11.3%)	30 (11.4%)	0.1 [−5.7; 5.8]	0%
*Anosmia*	29 (6.3%)	11 (5.6%)	18 (6.8%)	1.1 [−3.3; 5.3]	0%
*Visual disorders*	13 (2.8%)	8 (4.1%)	5 (1.9%)	−2 [−5.2; 0.9]	0%
*Dizziness*	10 (2.2%)	5 (2.6%)	5 (1.9%)	−0.6 [−3.3; 1.9]	0%
*Psychotic manifestations*	10 (2.2%)	4 (2%)	6 (2.3%)	0.2 [−2.4; 2.6]	0%
*Myoclonus*	9 (2%)	0	9 (3.4%)	2.6 [0.7; 4.9]	0%
*Ataxia*	6 (1.3%)	5 (2.6%)	1 (0.4%)	−1.6 [−4; 0.1]	0%
Patient outcomes	*N* = 424	*N* = 176	*N* = 248		
*Death*	45 (10.6%)	15 (8.5%)	30 (12.1%)	3.5 [−2.1; 9.1]	1.2%
*Hospitalized*	58 (13.7%)	15 (8.5%)	43 (17.3%)	8.7 [2.6; 14.7]	33.5%
*Rehabilitation*	148 (34.9%)	31 (17.6%)	117 (47.2%)	29.4 [21.1; 37.5]	100%
*Hospital discharge*	173 (40.8%)	115 (65.4%)	58 (23.4%)	−39.8 [−48.4; −30.8]	100%

Note: A difference in absolute value greater than 10% (highlighted in bold) indicates clinical significance

The most frequent neurological symptoms leading to an MRI scan were confusion (40%), consciousness disorders (35.8%), acute onset weakness or aphasia (27.5%), agitation (20%), and difficulties weaning from sedation (19.2%).

Patients in ICU care compared to patients who were not admitted to the ICU were more likely to require oxygen supplementation (98.9% vs. 45.1%), to be intubated (79.5% vs. 4.1%), to present with ARDS (89.4% vs. 14.9%), to suffer from consciousness disorders (48.3% vs. 19%), and to experience difficulties weaning from sedation (31.9% vs. 2.1%). Patients admitted to the ICU more frequently required rehabilitation after discharge (44.5% vs. 15.9%) and were less frequently directly discharged to home (22.1% vs. 59%).

Regarding patient outcomes, categorized as death or home discharge, oxygen supplementation, intubation, ARDS, consciousness disorders, and difficulties weaning from sedation were associated with a lower probability of home discharge and a higher mortality rate (Table 2).

**Table 2:** Patient outcomes

	Outcome		Proportion difference with 95% Bayesian credible interval	Probability that the difference is > 10%
	Death	Home discharge		
Sex, male	29 (64.4%)	112 (64.7%)	−0.3 [−15.9; 14.3]	10.8%
Inpatients (at the time of MRI)	45 (100%)	159 (91.9%)	5.9 [0.8; 10.7]	4.2%
Respiratory status				
*Oxygen supplementation*	41 (91.1%)	96 (55.5%)	33.3 [21.4; 43.5]	100%
*Orotracheal intubation*	26 (57.8%)	38 (22%)	34 [18.6; 49.1]	99.9%
*ECMO*	1 (2.2%)	2 (1.2%)	0.7 [−1.8; 5.3]	0.2%
*ARDS*	31 (68.9%)	57 (32.9%)	34.2 [18.8; 48.4]	99.9%
Neurological symptoms				
*Confusion*	10 (22.2%)	69 (39.9%)	−16.7 [−29.7; −2.3]	83%
*Consciousness disorders*	25 (55.6%)	38 (22%)	31.8 [16.4; 46.8]	99.7%
*Acute onset weakness or aphasia*	6 (13.3%)	50 (28.9%)	−14.4 [−25.2; −2]	78%
*Agitation*	6 (13.3%)	31 (18%)	−4.2 [−14.2; 7.7]	14.7%
*Difficult weaning from sedation*	16 (35.6%)	13 (7.5%)	25.8 [12.7; 40.2]	99.2%
*Headaches*	2 (4.4%)	27 (15.6%)	−9.7 [−16.9; −1.2]	48.9%
*Seizures*	9 (20%)	21 (12.1%)	7.2 [−3.6; 20]	30.8%
*Anosmia*	2 (4.4%)	10 (5.8%)	−0.9 [−6.4; 6.5]	0.1%
*Visual disorders*	1 (2.2%)	6 (3.5%)	−0.7 [−4.6; 4.7]	0%
*Dizziness*	0	5 (2.9%)	−1.4 [−4.4; 2.2]	0%
*Psychotic manifestations*	0	3 (1.7%)	−0.6 [−2.9; 2.2]	0%
*Myoclonus*	1 (2.2%)	3 (1.7%)	0.5 [−2.5; 5.3]	0.2%
*Ataxia*	0	3 (1.7%)	−0.6 [−2.9; 2.2]	0%

Note: A difference in absolute value greater than 10% (highlighted in bold) indicates clinical significance

### Imaging findings

**Fig. 3:**
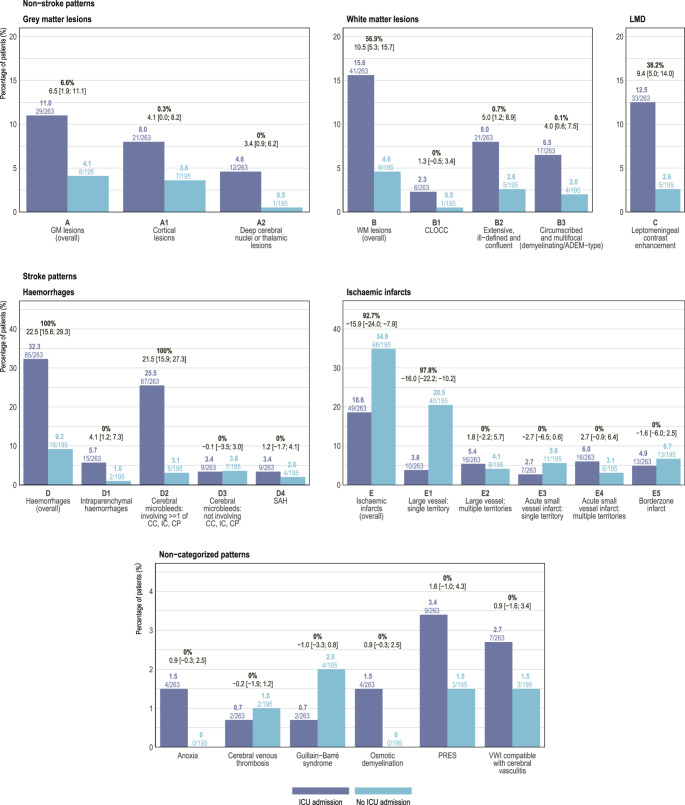
Bar plot illustrating the frequency of imaging findings categorized according to the proposed rating framework, stratified by ICU admission and non-ICU admission (displayed as both raw counts and percentages). Results from the Bayesian analysis are displayed above each paired bar, with the difference in absolute value in bold and the difference in proportion with the corresponding 95% Bayesian credible interval. Difference in absolute value greater than 10% indicate clinical significance. LMD, Leptomingeal disease

**Fig. 4:**
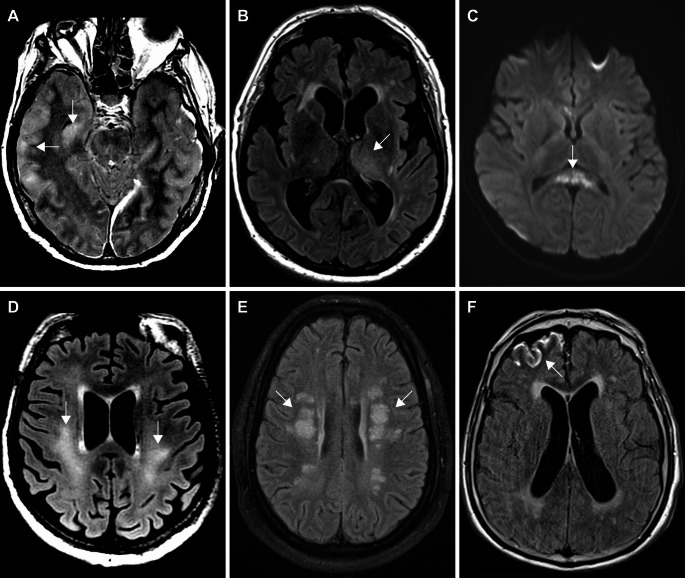
Non-stroke patterns: Axial FLAIR (**a**, **b**, **d**, **e**), post-contrast axial FLAIR (**f**), axial diffusion-weighted (**c**) MR images. A: cortical lesions involving the right hippocampus and right lateral temporal cortex lesions (arrowheads; pattern A1); b: left thalamic lesion (arrowhead; pattern A2); c: Cytotoxic lesion of the corpus callosum (CLOCC) (arrowhead; pattern B1), showing restricted diffusion; d: extensive, ill-defined, and confluent supratentorial white matter lesions (arrowheads; pattern B2); e: circumscribed and multifocal white matter lesions (arrowheads; pattern B3); f: right frontal leptomeningeal enhancement (arrowhead; pattern **C**)

**Fig. 5:**
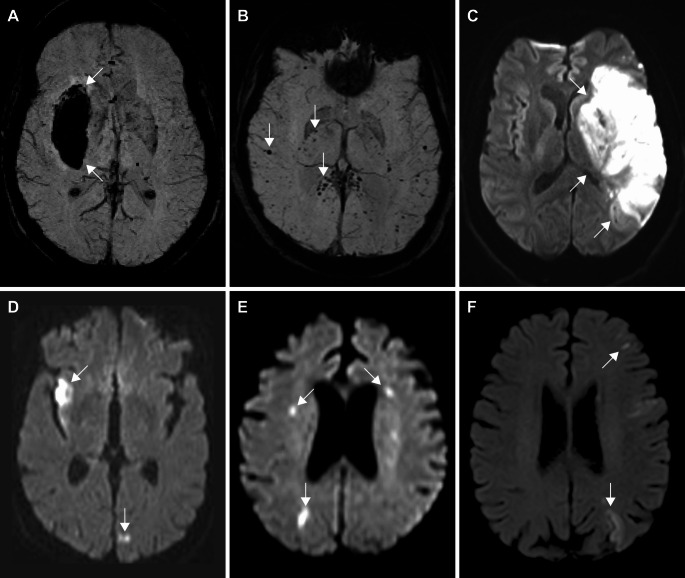
Hemorrhagic and ischemic patterns: SWI (**a**, **b**), axial diffusion (**c**-**f**) weighted MR images. A: intraparenchymal hematoma (arrowhead; pattern D1), b: cerebral microbleeds with involvement of internal capsules, corpus callosum splenium and sub-cortical white matter (arrowheads; pattern D2), c: left middle cerebral artery infarct: single territory large vessel infarct (arrowhead; pattern E1), d: multiple territories large vessel infarcts (arrowheads; pattern E2), e: multiple acute small vessel infarcts (arrowheads; pattern E4), f: border zone infarcts (arrowheads; pattern E5)

Overall, 41.5% of patients had normal MRI scans or non-Covid-19–related abnormalities detected on prior imaging. Detailed imaging findings and pattern categorization across ICU and non-ICU patients are shown in Fig. 3. Representative imaging patterns are shown in Figures 4 and 5.

Ischemic infarct patterns (E) were the most prevalent overall, with at least one such pattern seen in 117 patients (25.6%) and were more frequently observed in non-ICU patients (34.9%) than in ICU patients (18.6%). Within this category, large vessel infarcts were the most frequently overserved pattern, described in 74 patients (16.2%), of which in 50 patients either a single (E1) or in 24 multiple (E2) vascular territories were involved. Of 40 patients with acute DWI-positive small vessel infarcts, 18 were single- and 22 multifocal (E3 and E4, respectively). Although ischemic lesions were overall more prevalent in non-ICU patients, those involving multiple large vessel territories and multifocal DWI-positive lesions were slightly more frequent in ICU patients.

**Fig. 6:**
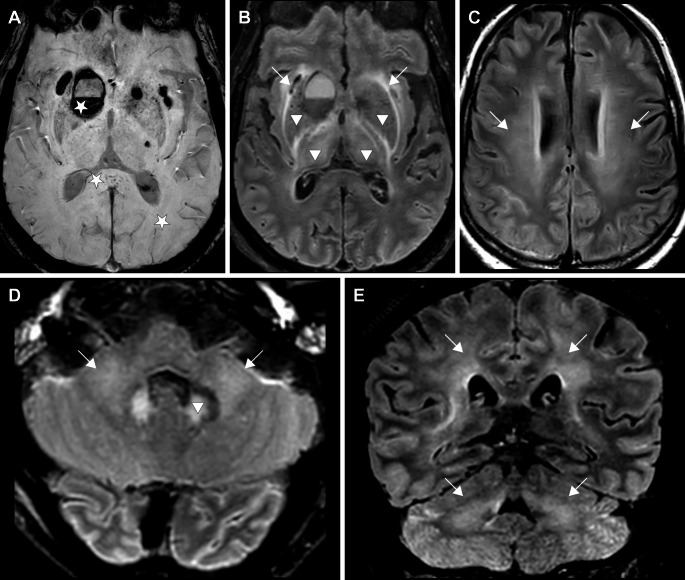
70-year-old man with a SARS-CoV–induced acute respiratory distress syndrome, requiring mechanic ventilation, experiencing difficulties weaning from sedation and confusion. Axial SWI (**a**), axial FLAIR (**b**, **c**, **d**), coronal FLAIR (**e**) weighted MR images. SWI hypointensities likely represent microbleeds (**a**, star) and intraparenchymal hematomas (**a**, arrowhead). Deep cerebral nuclei, thalamic and dentate nuclei FLAIR hyperintensities (**b**, **d**; arrowheads). Extensive, ill-defined, and confluent white matter FLAIR hyperintensities in subcortical, periventricular, and middle cerebellar peduncle regions (**b**, **c**, **d**, **e**; arrows)

Haemorrhagic lesions overall (D) were more frequent in ICU patients (85 patients, 32.3% vs. 18 patients, 9.2%) and the most frequent pattern were cerebral microbleeds found in 73 patients (15.9%), with microbleeds involving the corpus callosum, internal capsules, cerebellar peduncles (D2) were more frequent in ICU patients (25.5% vs. 3.1%). No difference regarding microbleeds not involving these locations (D3) has been found (3.4% vs. 3.6%). Macroscopic intraparenchymal haemorrhage (D1) was observed in 17 patients (3.7%). MRI scans from a representative patient with combined susceptibility and FLAIR abnormalities are shown in Figure 6.

Overall, white matter lesions (B) were more frequent than grey matter lesions (A). The most frequent white matter pattern seen in 26 patients (5.7%) were extensive, ill-defined, and confluent lesions (B2), either infra- or supratentorial. The most frequent grey matter pattern were cortical lesions (A1; 28 patients, 6.1%). Leptomeningeal contrast enhancement (C) was observed in 38 patients (8.3%) and in 38/150 (25.3%) patients with post-gadolinium FLAIR imaging. White matter lesions and leptomeningeal contrast enhancement overall were more commonly observed in ICU patients compared to non-ICU patients (15.6% vs. 4.6% and 12.5% vs. 2.6%, respectively).

In summary, our proposed standardized framework proved highly effective, capturing all identified lesions and imaging abnormalities in 430 of 458 patients (94%). Of them, 145 patients had an MRI scan showing at least one predefined pattern, while 63, 32, and 11 patients showed two, three, and four patterns, respectively, and in 1 patient seven patterns were observed. The most common additional imaging findings not covered by our proposed classification, observed in 28 patients (8.7%), were posterior reversible encephalopathy syndrome (PRES; 12 patients), arteritis (10 patients), and Guillain–Barré syndrome (6 patients).

### Patient outcomes

Patient outcomes did not differ substantially across MRI patterns, with proportion differences under 10% in absolute value (eTable 4).

## Discussion

Our study represents a large retrospective analysis of MR brain imaging findings in patients with acute Covid-19 during the first pandemic wave, including 458 patients with neurological symptoms. Previously published series consisted of an average of 39 patients (range [11–115], eTable 2). To analyse the MR images, we used a novel, standardized framework of specific MR imaging patterns, which was developed by international expert consensus and a review of the literature.

The proposed framework of MR imaging patterns has been shown to be efficient and comprehensive, covering cerebral imaging findings seen in 252 of 268 patients (94%) with confirmed Covid-19, neurological symptoms and an abnormal MRI scan. Among the imaging patterns not captured by the proposed criteria, the most common findings were PRES and vasculitis. Acute necrotizing encephalopathy, a rare but severe complication of Covid-19 and other viral infections linked to intracranial cytokine storms and documented by other groups, was not observed in our cohort [24]. Consequently, the current version of our framework should be updated by including these two patterns.

Ischemic stroke was the most frequent imaging pattern (25.6%) and was overall more prevalent in non-ICU patients (34.9%) compared to ICU patients (18.6%), which likely reflects the fact that patients with ischemic strokes are typically admitted to the stroke unit rather than the ICU. An exception to this was the subgroup of patients with multiple territory infarcts.

Large vessels strokes were more common than small vessel, lacunar strokes and included multi-territory infarcts, consistent with previously published data [21, 25, 26]. SARS-CoV-2 infection seems to be an independent risk factor of ischemic stroke because of a hypercoagulable state with an elevation of d-Dimer, other coagulation markers and systemic inflammation with possible cardiac and arterial wall involvement [27–30]. Although our study did not include a control group, a UK multicentre case–control study comparing data of 86 strokes with evidence of Covid-19 at the time of onset with 1,384 Covid-19–negative strokes during the same time found Covid-19–positive strokes more likely to involve multiple large vessels, to be more severe and to be associated with higher d-Dimer level [31]. The marginally increased frequency of both large- and small-vessel multi-territory infarcts in ICU patients may reflect the severity of the underlying disease, while critical illness–induced inflammatory mechanisms could exacerbate and extend the ischemic process.

Haemorrhagic intracranial lesions – intraparenchymal, subarachnoid, and cerebral microbleeds – were observed in 22.5% of our patients and were more frequent in ICU patients (32.3% vs. 9.2%). A higher mortality rate has been found in patients with Covid-19–related intracranial haemorrhage [32, 33]. Patients with severe Covid-19 received either prophylactic anticoagulation because of a prothrombotic state or therapeutically for pulmonary embolism or venous thrombosis, increasing the risk of intracranial haemorrhage [33, 34].

The most frequent haemorrhagic patterns were microbleeds involving the corpus callosum, the internal capsules and the cerebellar peduncles, differentiating from the anatomical localization of microbleeds seen in amyloid angiopathy or vascular risk factor-associated (“hypertensive”) arteriolosclerosis. The pattern was more frequent in ICU patients (25.5% vs. 3.1%).

It is important to note that what is typically referred to as cerebral microbleeds in the imaging literature may not always correspond to true microbleeds at the histopathological level. Instead, it can encompass a range of conditions, such as intravascular microthrombi or areas of stagnant blood flow rich in deoxyhaemoglobin within small arteries and veins [35].

The underlying mechanisms of these focal susceptibility artifacts, commonly referred to as microbleeds, remain incompletely understood, and several possible explanations have been proposed [13, 36, 37]: one hypothesis is that the observed microhaemorrhages may reflect thrombotic microangiopathy, a condition seen in various organs affected by SARS-CoV-2 infection, particularly in the lungs [38] as well as in neuropathological studies of the brain [39, 40]. Another possibility is that kidney failure might contribute to these findings by increasing the permeability of the blood–brain barrier due to elevated concentrations of uremic toxins [13]. A third hypothesis proposes that the microbleeds may be secondary to hypoxemia [13, 36, 37]. Similar microhaemorrhages have been observed in critically ill patients and in those experiencing high-altitude oedema, where oxygen deprivation may lead to disruption of the blood–brain barrier and subsequent extravasation of erythrocytes [41, 42]. Furthermore, SWI-detected microbleeds, also called “critical-illness associated microbleeds”, have been reported in patients receiving mechanical ventilation, extracorporeal membrane oxygenation (ECMO), and other forms of assisted oxygenation, both in imaging studies and at autopsy [43–45]. However, in our cohort, only a small group of patients (2.4%) required ECMO.

After ischemic strokes and haemorrhages, white matter lesions were the third most common imaging pattern, observed in 10.9% of cases, followed by grey matter lesions at 8.1%. These results are consistent with previously published data (eTable 2). The most frequent white matter pattern were extensive, ill-defined and confluent lesions (5.7%). It has been hypothesized that these signal abnormalities could be secondary to hypoxemia, as described in post-hypoxic delayed leukoencephalopathy [46–49]; in another study, the confluent white matter hyperintensities were related to acute renal failure, hypernatremia, anaemia, and obesity rather than hypoxia [50]. Furthermore, there is evidence that prolonged mechanical ventilation can induce brain damage by several mechanisms [51].

Among grey matter lesions, cortical involvement was more common than involvement of deep cerebral nuclei. Cortical mesial temporal lobe lesions have been frequently reported with a pattern mirroring the radiological appearances of limbic encephalitis [52, 53]. Involvement of the temporal lobe has, however, also been described secondary to mechanical ventilation [54]. Furthermore, the mesial temporal lobe changes could reflect post-infectious inflammatory and immune-mediated processes rather than direct viral invasion. This is supported by the fact that the SARS-CoV-2 virus is rarely found in the CSF and neuropathological samples (eTable 1) [15].

Similar considerations apply to leptomeningeal contrast enhancement. An inflammatory aetiology is supported by neuropathological studies of patients with Covid-19, which report leptomeningeal lymphocytic infiltration (eTable 1). A similar pattern has also been described in multiple sclerosis, where perivascular leptomeningeal inflammatory infiltrates can be found [55].

We did not identify any specific MRI pattern associated with patient outcomes, as measured by either death or discharge to home. Most patients were in ICU care (57.4%), requiring oxygen supplementation (76%), with ARDS (57.4%), and the overall prognosis was related to respiratory failure rather than cerebral MRI abnormalities.

Our study has some limitations, mainly related to its retrospective design, that need to be acknowledged. First, over half of the patients were in ICU care, which may result in selection bias. This is because, during the first wave of the pandemic, many centres restricted MRI examinations of Covid-19 patients to those deemed clinically essential, and with a potential immediate impact on patient care. Consequently, patients with relatively mild neurological symptoms – such as headaches, anosmia, visual disorders, dizziness – were less frequently imaged during the first wave. Furthermore, this study does not include patients suffering from “long covid”, a condition primarily recognized after our data collection.

The use of CT or MRI depends on local practice and policy decisions. During the first wave of the pandemic, some centres used CT as their only imaging modality, aiming to keeping their MR systems “Covid free.” As a result, the present study may underestimate the incidence of certain cerebral abnormalities detectable by CT, such as ischemic stroke and intracranial haemorrhage, since only patients who underwent MRI were included. On the other hand, MRI allows for the detection of smaller acute lesions and multi-territory infarcts. MRI allows the visualization of microhaemorrhages that would not be detected with by CT, and their frequency would be underestimated in any studies including CT scans.

Secondly, there was often some delay between the MRI and the onset of, mainly respiratory, Covid-19 symptoms (22.8 ± 18.9 days). In ICU patients, indications for MRI scans frequently included confusion, consciousness disorders, agitation, and difficulties weaning from sedation. Considering that these patients were sedated, the precise timing of the onset of neurological symptoms could not always be accurately determined.

Finally, the retrospective, multicentre design of the study limited the ability to standardize MRI acquisition protocols. Core clinical MRI sequences were available in all patients, and contrast-enhanced MRI images were acquired in 308 patients, of which 150 cases also included post-contrast FLAIR images, which is the most sensitive sequence for detecting leptomeningeal contrast enhancement in infectious, neoplastic, and inflammatory diseases [55]. Although leptomeningeal contrast enhancement was already one of the most frequently observed imaging patterns, the lack of systematic acquisition of contrast-enhanced FLAIR images is likely to underestimate its incidence.

In summary, this is, to our knowledge, the largest cohort study of brain MRI in patients with confirmed acute Covid-19 infection. We analysed MRI scans of 458 patients from five different countries using a novel, standardized classification framework according to predefined specific imaging patterns developed by a consortium of experts, which covered 94% of observed abnormalities.

In non-ICU patients, ischemic strokes were the most common imaging abnormalities, while in patients admitted to the ICU haemorrhagic lesion were the most frequent imaging findings, particularly cerebral microbleeds involving the corpus callosum, internal capsules, and cerebellar peduncles. Our proposed structured framework proved highly pertinent to capturing the various neuropathological disease processes associated with Covid-19 and could in the future facilitate a standardized reporting of brain MRI findings in acute Covid-19.

## Supplementary information

Below is the link to the electronic supplementary material.

Supplementary Material 1 (PDF 158 KB)

## Data Availability

Anonymized data can be made available upon reasonable request by any qualified investigator for the purpose of replicating procedures and results. Requests should be directed to Prof. Stéphane Kremer via e-mail (stephane.kremer@chru-strasbourg.fr).

## Abbreviations

ADEM: Acute disseminated encephalomyelitis
ARDS: Acute respiratory distress syndrome
CLOCC: Cytotoxic lesion of the corpus callosum
ECMO: Extracorporeal membrane oxygenation
RT-PCR: Reverse transcriptase-polymerase chain reaction
ICU: Intensive care unit
PRES: Posterior reversible encephalopathy syndrome
