# Supplementary material for: Structured assessment of brain MRI in Covid-19–related neurological disease: an international multicentre study
Source: Neuroradiology. 2025 Sep 26;67(11):3121–36. doi: 10.1007/s00234-025-03787-8 (PMC12743100; doi:10.1007/s00234-025-03787-8)
Supplement: Supplementary file 1 — Supplementary Material 1 (PDF 158 KB) [file 234_2025_3787_MOESM1_ESM.pdf]

Structured assessment of brain MRI in Covid-19–related neurological disease: An international multicentre study

eMethods literature search

Study selection

Relevant articles were assessed for eligibility by two independent investigators after full-text evaluation based on the following criteria:

Inclusion criteria: (i) studies published in English; (ii) patients with Covid-19 assessed by positive RT-PCR for SARS-CoV-2; for non-stroke intracerebral abnormalities: (iii) study populations consisting of at least ten patients who underwent cerebral MRI; (iv) studies with neuropathological data.

Exclusion criteria: (i) studies published in another language than English; (ii) reviews, meta-analyses, and commentaries; for non-stroke intracerebral abnormalities: (iii) studies with less than ten patients; (iv) studies without cerebral MRI or with insufficient MRI data description; (v) study population completely overlapping with that of other studies; (vi) studies reporting patients with non-Covid-19–related abnormalities only.

Data extraction

Two reviewers performed the data extraction independently. Disagreements and inconsistencies were resolved by a third author.

The main information extracted for non-stroke intracerebral abnormalities included: name of first author, journal name, country, study design: single or multicentre, number of patients with MRI, number of MRI without Covid-19–related abnormality, number of patients with grey matter, hippocampus, cortex, bilateral thalami, deep cerebral nuclei, white matter, splenium of the corpus callosum, cerebellar peduncles, white matter circumscribed or extensive abnormalities, PRES, white matter microhaemorrhages, leptomeningeal, cranial nerve, vessel wall contrast enhancement, olfactory bulb abnormalities.

The main information extracted for neuropathological studies included: name of first author, journal name, country, number of patients, number of patients with neuropathology, number of patients with histology, white matter microhaemorrhages, white matter axonal injury, perivenular myelin loss, neocortical infarcts, large infarcts, white matter infarcts, deep grey matter infarcts, leptomeningeal lymphocytic inflammation, perivascular lymphocytic inflammation, microthrombi, endotheliitis, confirmed case of SARS-CoV-2.

eTable 1: Neuropathological studies

First author	Journal	Country	Patients	Neuropathology	Histological analysis	Large haemorrhages	WM microhaemorrhages	WM axonal injury	Perivenular myelin loss	Neocortical infarcts	Large infarcts	WM infarcts	Deep GM infarcts	Leptomeninges lymphocytic inflammation	Perivascular Lymphocytic inflammation	Microthrombi	MG brainstem activation	Endotheliitis	SARS-CoV-2 evidence (brain)
Al-Dalahmah O. et al.	Acta Neuropathol	USA	1	1	1	1					1			1	1				0
Bradley BT. et al.	Lancet	USA	14	5	5		1	0	0	0	0	0	0	0	0	0		0	0
Bryce C. et al.	medRxiv	USA	67	23	20		+	+	0	3	1	3	1	0	2	6		0	0
Conklin J. et al.	medRxiv	USA	16	1	1	0	1	1	1			1							0
Deigendesch N. et al.	Acta Neuropathol	Switzerland	7	7	7	0	0	0	0	0	0	0	0	0	0	0	7	0	4
Fabbri V. P et al.	Brain Pathol	Italy	10	10	10				10	10		10	10	1	1	10			1
Hanley B. et al.	Lancet Microbe	UK	10	5	5					5	1	5			5				1
Jaunmuktane Z. et al	Acta Neuropathol	UK	2	2	2	0	1	1	0	1	1	1	2	1	0	1		0	0
Kantonen J. et al.	Brain Pathol	Finland	4	4	4		1	1	1	0	0	1	0	0	0	0		0	0
Kirschenbaum D. et al.	Lancet	Switzerland	2	2	2										2	2			0
Matschke J. et al.	Lancet Neurol	Germany	43	43	43						6			34	37				21
Reichard RR. et al.	Acta Neuropathol	USA	1	1	1	0	1	1	1	1	0	1	0	0	1	0		0	0
Remmelink M. et al.	Crit Care	Belgium	17	11	11	1	8	0	0	0	3	0	0	0	0	0		0	9
Schaller T. et al.	JAMA	Germany	10	10	10		0	0	0	?	0	0	0	0	0	0		0	0
Schurink B. et al.	Lancet Microbe	Nederland	21	9	9		0	0	0	0	0	0	0	9	9	0		0	0
Solomon IH. et al.	N Engl J Med	USA	18	18	18									1	2				5
von Weyhern CH. et al.	Lancet	Germany	6	6	6		4	6	0	?	0	0	0	6	6	0		0	0

GM: Grey matter; WM: White matter

eTable 2: Neuroradiological studies

First author	Journal	Study design	Country	Patients with MRI (normal)	Main neuroimaging findings
Agarwal S. et al.	Stroke	Single centre	USA	115 (33)	30 with WM lesions (18 extensive, ill-defined and confluent WM FLAIR hyperintensities) and 25 with disseminated WM microhaemorrhages
Anzalone N. et al.	J Neurol	Single centre	Italy	21 (17)	4 with GM lesions (cortex involvement) including one with intracerebral enhancement
Chetrit A. et al.	J Infect	Single centre	France	23 (4)	19 with olfactory bulb abnormalities
Chougar L. et al.	Radiology	Single centre	France	73 (30)	4 with GM lesions (deep cerebral nuclei), 8 with WM lesions, one with leptomeningeal enhancement, 2 with cranial nerve enhancement, and 4 with intracerebral enhancement
Conklin J. et al.	medRxiv	Single centre	USA	16 (3)	11 with WM microhaemorrhages
Coolen T. et al.	Neurology	Single centre	Belgium	19 (11)	4 with olfactory bulb abnormalities, 2 with WM microhaemorrhages and one with posterior reversible encephalopathy syndrome
Freeman CW. et al.	AJR	Single centre	USA	59 (?)	6 with WM lesions and 4 with WM microhaemorrhages
Helms J. et al.	NEJM	Single centre	France	13 (0)	8 with leptomeningeal enhancement
Kandemirli SG et al.	Radiology	Multicentre	Turkey	27 (15)	10 with GM lesions (cortex involvement), 3 with ill-defined and confluent WM FLAIR hyperintensities, and 5 with leptomeningeal enhancement
Klironomos S. et al.	Radiology	Single centre	Sweden	43 (?)	19 with WM lesions (including 7 patients with an involvement of the cerebellar peduncles, and 18 cases with ill-defined and confluent WM FLAIR hyperintensities), and 29 with WM microhaemorrhages
Kremer S. et al.	Radiology	Multicentre	France	37 (?)	18 with GM lesions (including 16 with an involvement of the mesial temporal lobes), and 17 with WM lesions (including 13 cases with circumscribed and multifocal WM FLAIR hyperintensities)
Lin J. et al.	AJNR	Multicentre	USA	51 (25)	2 with posterior reversible encephalopathy syndrome, and 2 with cranial nerve enhancement
Paterson RW. et al.	Brain	Multicentre	UK	43 (11)	5 with GM lesions and 10 with WM lesions
Radmanesh A. et al.	AJNR	Single centre	USA	35 (?)	
Radmanesh A. et al.	Radiology	Single centre	USA	11 (?)	10 with WM lesions (including 4 patients with an involvement of the cerebellar peduncles, and 10 cases with ill-defined and confluent WM FLAIR hyperintensities),
Sawlani V. et al.	Clin Radiol	Single centre	UK	36 (16)	5 with WM lesions (including 4 patients with circumscribed and multifocal WM hyperintensities), and 12 with WM microhaemorrhages
Yoon BC et al.	AJNR	Single centre	USA	52 (?)	7 with WM lesions and 7 with WM microhaemorrhages

FLAIR: Fluid attenuated inversion recovery; GM: Grey matter; WM: White matter

eTable 3: Number of cases included per centre

Country	Centre	N	Experience reader 1 (years)	Experience reader 2 (years)
France	Site 1	91	20	9
	Site 2	18	20	9
	Site 3	15	20	9
	Site 4	11	20	9
	Site 5	10	20	9
	Site 6	10	20	9
	Site 7	6	20	9
	Site 8	5	20	9
	Site 9	5	20	9
	Site 10	4	20	9
	Site 11	3	20	9
	Site 12	3	20	9
	Site 13	2	20	9
	Site 14	1	20	9
	Site 15	1	20	9
	Site 16	1	20	9
	Site 17	1	20	9
	**Sub-total**	**18 (40.9%)**		
Spain	Site 1	44	30	5
	Site 2	31	30	14
	Site 3	24	28	26
	**Sub-total**	**99 (21.6%)**		
Italy	Site 1	61	25	2
	Site 2	18	30	30
	**Sub-total**	**79 (17.2%)**		
UK	Site 1	36	12	3
	Site 2	18	30	15
	**Sub-total**	**54 (11.8%)**		
Brazil	Site 1	39	21	18
	**Sub-total**	**39 (8.5%)**		
	**Total**	**458 (100%)**		

eTable 4: Neuroimaging patterns and patient outcome

Imaging pattern	Death	Home discharge	Proportion difference with 95% Bayesian credible interval	Probability that the difference is >10%
A1	4 (8.9%)	7 (4.1%)	4 [-2.4; 13.2]	8%
A2	1 (2.2%)	3 (1.7%)	0.5 [-2.5; 5.4]	0.1%
B1	1 (2.2%)	2 (1.2%)	0.7 [-1.8; 5.3]	0.1%
B2	4 (8.9%)	7 (4.1%)	4 [-2.5; 13.1]	7,9%
B3	2 (4.4%)	2 (1.2%)	2.2 [-1.2; 8.3]	1.1%
C	5 (11.1%)	9 (5.2%)	5.1 [-2.4; 15.3]	14,2%
D1	4 (8.9%)	4 (2.3%)	5.2 [-0.7; 14.2]	11.5%
D2	9 (20%)	23 (13.3%)	6.2 [-4.8; 19.2]	25.8%
D3	4 (6.7%)	7 (4.1%)	2.2 [-3.5; 10.4]	3%
D4	3 (6.7%)	3 (1.7%)	3.7 [-1.1; 11.4]	4.4%
E1	6 (13.3%)	22 (12.7%)	0.6 [-8.9; 12.2]	5%
E2	3 (6.7%)	6 (3.5%)	2.6 [-2.9; 10.7]	3.4%
E3	1 (2.2%)	9 (5.2%)	-2.1 [-6.6; 3.9]	0.1%
E4	6 (13.3%)	7 (4.1%)	7.9 [0; 18.6]	30.2%
E5	4 (8.9%)	7 (4.1%)	4 [-2.4; 13.1]	8%
Anoxia	3 (6.7%)	0	3.9 [0.3; 10.9]	3.8%
Cerebral venous thrombosis	0	1 (0.6%)	0 [-1.2; 1.8]	0%
Guillain–Barré syndrome	0	2 (1.2%)	-0.3 [-2.1; 2.2]	0%
Osmotic demyelination	3 (6.7%)	0	4 [0.3; 10.9]	3.8%
PRES	1 (2.2%)	1 (0.6%)	0.9 [-1.2; 5.1]	0.1%
VWI compatible with cerebral vasculitis	3 (6.7%)	2 (1.2%)	3.9 [-0.5; 11.4]	4.6%
